# Clinical Characteristics of Patients and Whole Genome Sequencing-Based Surveillance of *Escherichia coli* Community-Onset Bloodstream Infections at a Non-tertiary Hospital in CHINA

**DOI:** 10.3389/fmicb.2021.748471

**Published:** 2021-10-07

**Authors:** Fenghong Chen, Tao Lv, Yupeng Xiao, Aizhi Chen, Yonghong Xiao, Yunbo Chen

**Affiliations:** ^1^Clinical Laboratory, The First Hospital of Putian City, Putian, China; ^2^State Key Laboratory for Diagnosis and Treatment of Infectious Diseases, The First Affiliated Hospital, Zhejiang University School of Medicine, Hangzhou, China; ^3^Collaborative Innovation Center for Diagnosis and Treatment of Infectious Diseases, Hangzhou, China

**Keywords:** *Escherichia coli*, community-onset blood stream infection, whole-genome sequencing, epidemiology, virulence genes

## Abstract

**Background:** *Escherichia coli* is the most common pathogens in patients with community-onset blood stream infections (COBSI). Knowledge of the epidemiology of this disease is crucial to improve allocation of health resources, formulate isolation strategies that prevent transmission, and guide empirical antibiotic therapy.

**Methods:** This retrospective observational study examined patients with *E. coli* COBSI (EC-COBSI) at a non-tertiary hospital in China. Whole-genome sequencing and analysis of the isolates was performed. The relationships of clinical variables with antimicrobial resistance and the genetic background of the isolates were examined.

**Results:** There were 148 isolates in patients with EC-COBSI. All isolates were susceptible to ceftazidime/avibactam, carbapenems, and tigecycline; 35.1% were positive for extended spectrum β-lactamase (ESBL+); and *bla_*CTX*__–__*M*__–__14_* was the most common ESBL gene. Patients with ESBL- isolates were more likely to receive appropriate empiric treatment than those with ESBL+ isolates (61.5% vs. 91.4%, *p* < 0.001), but these two groups had similar mortality rates. The overall 30-day mortality rate was 9.5%. Phylogenetic analysis showed that the isolates were diverse, and that the main sequence types (STs) were ST95, ST131, and ST69. Intra-abdominal infection was the primary source of disease, and isolates from these patients had lower frequencies of virulence genes.

**Conclusion:** The mortality rate of patients with EC-COBSI was unrelated to ESBL status of the isolates. Most isolates had low resistance to most of the tested antimicrobial agents. The isolates were diverse, and multiple strains were related. Prevention and control of EC-COBSI should target prevention of patient colonization and the living environment.

## Introduction

The SENTRY program was established in 1997 to monitor the predominant bacterial pathogens and the antimicrobial resistance patterns of organisms isolated from patients with various types of infections, including bloodstream infection (BSI) (Diekema et al., 2000). The results indicated that *Escherichia coli* was the most common species isolated from patients with community-onset bloodstream infections (COBSI) (Diekema et al., 2019) since 2005, consistent with studies from other regions (Bou-Antoun et al., 2016), including China (Quan et al., 2017). *E. coli* COBSI (EC-COBSI) pose serious therapeutic challenges, and the use of inappropriate antimicrobial therapies is especially concerning because it can lead to an increased prevalence of antimicrobial resistance (AMR), long hospital stays, high healthcare costs, and substantial overall clinical impact (Lin et al., 2019). There are some reports that risk factors for EC-COBSI (Abernethy et al., 2017; Quan et al., 2017) and bacterial determinants, such as virulence factors and antimicrobial susceptibility, are related to the process of infection and disease severity (Mora-Rillo et al., 2015). Whole genome sequencing (WGS) provides important information on the spread of specific bacterial clones that are responsible for BSI (Pérez-Vázquez et al., 2019).

However, there is little known about the prevalence of *E. coli* clonal groups responsible for bacteremia and their associations with the clinical and demographic characteristics of patients in mainland China. Several previous studies focused on drug-resistant strains, such as extended spectrum β-lactamase (ESBL) -producing *E. coli* isolates (Quan et al., 2017), but this may have led to over- or underrepresentation of certain clonal groups. Additionally, most previous studies on this topic focused on patients in large tertiary care hospitals, and the epidemiological and clinical features of these patients may differ from those of patients with EC-COBSI who are in non-tertiary hospitals. Furthermore, a limitation of these previous studies is that they did not examine the relationships between clinical metadata and genes of the isolates.

We performed a retrospective observational study of EC-COBSI at a non-tertiary hospital in eastern China. We comprehensively examined the clinical data of source patients, characterized their antimicrobial resistance, used WGS to identify *E. coli* isolates, examined the population structure of the strains, determined the genetic types, and analyzed the molecular characteristics of all isolated strains. We also identified genes important in the main sequence types (STs) to better understand the reasons for their dominance. Our general purpose was to develop strategies that provide better control and prevention of antimicrobial resistance in EC-COBSI.

## Materials and Methods

### Study Population

This retrospective cohort study analyzed all *E. coli* clinical isolates from blood cultures at a local hospital that had approximately 1,200 beds. This hospital participates in the Blood Bacterial Resistant Investigation Collaborative System (BRICS) program in China, and maintains all pathogens from BSI patients for subsequent use in functional studies. Consecutive patients with monomicrobial *E. coli* infections were enrolled from 1 January 2018 to 31 December 2019. Isolates were excluded if they were from infants, repetitive strains, or had poor sequencing coverage. A total of 148 isolates were analyzed.

### Clinical Data

The medical records of all enrolled patients were examined to collect basic information, including demographic characteristics, residential address, and laboratory indicators, at the time when blood samples were taken for culture. The presence of comorbid conditions was also recorded. Information regarding empirical and targeted antibiotic treatments was documented, and patient outcomes were recorded.

### Definitions

*E. coli* bacteremia was defined by the presence of at least one bacterial culture that was positive for *E. coli*, and EC-COBSI by the presence of *E. coli* bacteremia within 48 h after hospital admission (Rodríguez-Baño et al., 2010). Appropriate antibiotic therapy was defined by the use of an antimicrobial agent to which the infectious organism had *in vitro* susceptibility (Huh et al., 2020). Cure was defined by resolution or abatement of clinical manifestations related to infection. Multi-drug resistance (MDR) was defined by resistance to three or more classes of antibiotics (Magiorakos et al., 2012).

### Microbiological Studies

Samples for aerobic and anaerobic blood cultures were collected and loaded into the BacT/ALERT^®^3D system according to the manufacturer’s recommendations. Immediate Gram staining and species identification was performed using the MicroScan WalkAwa**y** 96 plus System, following guidelines of the manufacturer.

### Antimicrobial Resistance Phenotyping

Susceptibility to 20 antimicrobial agents was determined according to Clinical and Laboratory Standards Institute (CLSI) guidelines (Clinical and Laboratory Standards Institute [CLSI], 2020). Minimum inhibitory concentrations (MICs) were interpreted according to CLSI guidelines, except for tigecycline and polymyxin B, which used breakpoints from the European Committee on Antimicrobial Susceptibility Testing (EUCAST).^1^ The MIC breakpoint of cefoperazone for Enterobacteriaceae was used to determine cefoperazone/sulbactam susceptibility, as described by Jones et al. (1987). Synergy tests were used to phenotypically identify isolates producing ESBL.

### Phylogenetic Analysis and Variant Detection

DNA extraction, sequencing, and assembly of reads were performed as described previously (Raven et al., 2016). Briefly, the raw Illumina reads were mapped to the *E. coli* reference genome EC958 (accession no. NZ_HG941718.1) using SAM tools mpileup (Li et al., 2009), and were assessed for single nucleotide polymorphisms (SNPs) using VarScan (Koboldt et al., 2013). Mobile genetic elements were masked and recombination filtration was performed using Gubbins (Croucher et al., 2015). Maximum likelihood (ML) phylogenetic trees were established using RAxML (Stamatakis, 2015) with the generalized time reversible (GTR) model and a gamma distribution to model site-specific rates of variation. Bootstrapping with 1,000 replicates was used to determine the robustness of the SNP-based ML phylogenetic tree. The minimum spanning trees (MST), based on cg-MLST data from all isolates, were generated using PHYLOViZ v2.0 with the geoBURST Full MST algorithm (Nascimento et al., 2017).

### Pangenome Analysis

Accessory genes were identified using Roary (Page et al., 2015), and accessory gene accumulation curves were generated separately for each dominant ST using R software. The relationships between the dominant STs and shared accessory genes were evaluated using non-metric multidimensional scaling (NMDS), and using the vegan package in R software (Oksanen et al., 2007). Variations of core gene and accessory gene content were assessed using pairwise distances. For each pair of isolates, the cophenetic distance from the core gene phylogenetic tree and the Jaccard distance were calculated. These two distances were plotted against each other to compare the diversity of accessory genes. Using assembled data, plasmid incompatibility types and subtypes were identified using PlasmidFinder (Carattoli et al., 2014).

### Genome-Wide Association Study

To further understand the characteristics of the dominant STs, a pangenome-wide association study was used to characterize pangenomic genes from the three main STs (ST95, ST131, and ST69) (Brynildsrud et al., 2016). The Kruskal-Wallis significance test with Bonferroni correction was used to confirm the significance of these relationships.

### Statistical Analysis

The characteristics of patients were expressed using proportions for categorical variables, and mean and standard deviation (SD) or median and interquartile range (IQR) for continuous variables. Pearson’s χ^2^-test was used for comparing categorical variables, Student’s *t*-test was used for comparing means, and the Mann-Whitney *U*-test was used for comparing medians. Multivariate analysis was performed using binary logistic regression. All statistical tests were 2-tailed, and a *P*-value of 0.05 or less was considered significant. Analyses were conducted using SPSS version 24.0 (SPSS Inc., Chicago, IL).

### Ethical Considerations

This work was approved by the Committee on Human Research, Publications and Ethics of the First Hospital of Putian City (No. 2021-004).

### Nucleotide Sequence Accession Numbers

PRJNA747133.

## Results

### Characteristics of Patients With *E. coli*-COBSI and *E. coli* Isolates

During the study period, 148 patients [54 men (36.5%) and 94 women (63.5%)] met our predefined study definition of EC-COBSI (Table 1). The males and females had similar demographics, except that more females had a history of diabetes mellitus (52.1% vs. 20.4%, *p* < 0.001) and more males had solid tumors (25.9% vs. 8.5%, *p* = 0.004). The most common source of bacteremia was an intra-abdominal infection (IAI), and this condition was more common in males than females (66.0% vs. 23.4%). The second-most common source was urinary tract infection (UTI), and this was more common in females than males (40.4% vs. 11.3%). A total of 80.7% of the patients received appropriate empirical treatment, and 56.9% received antibiotic monotherapy. β-lactam and β-lactamase inhibitor combinations (BLBLICs) were the most common empirical therapy, followed by fluoroquinolones and third-generation cephalosporins. Combination therapy was more common for patients receiving targeted therapy. The overall mortality rate at 30 days was 9.5%, and was similar in males and females (*p* = 0.6028).

**TABLE 1:** Demographic and clinical characteristics of patients with *E. coli* community-onset bacteremia.

**Variable**	**Total patients (*N* = 148)**	**Males (*n* = 54)**	**Females (*n* = 94)**	***p***	**ESBL- (*n* = 96)**	**ESBL+ (*n* = 52)**	***p***
Gender							0.279
Male	54 (36.5)	−	−		32 (33.3)	22 (42.3)	
Female	94 (63.5)	−	−		64 (66.7)	30 (57.7)	
Age, years	68.0 (56.8, 78.3)	70.0 (60.0, 77.5)	67.0 (55.0, 78.8)	0.791	68.5 (55.0, 78.0)	66.0 (57.0, 79.3)	0.753
Residency				0.1149			0.399
Urban	26 (17.6)	13 (24.17)	13 (13.8)		15 (15.6)	11 (21.2)	
Rural	122 (82.4)	41 (75.9)	81 (86.2)		81 (84.4)	41 (78.9)	
Within 4 weeks before admission							
Hospitalization	6 (4.1)	1 (1.9)	5 (5.3)	0.5507	4 (4.2)	2 (3.9)	1.000
Antibiotic exposure	5 (3.4)	1 (1.9)	4 (4.3)	0.759	3 (3.1)	2 (3.9)	1.000
Underlying disease							
Chronic kidney disease	7 (4.7)	2 (3.7)	5 (5.3)	0.965	5 (5.2)	2 (3.9)	1.000
Diabetes mellitus	60 (40.5)	11 (20.4)	49 (52.1)	<0.001	44(45.8)	16 (30.8)	0.075
Chronic obstructive Pulmonary disease	2 (1.4)	1 (1.9)	1 (1.1)	1	1 (1.0)	1 (1.9)	1.000
Solid tumor	22 (14.9)	14 (25.9)	8 (8.5)	0.004	11 (11.5)	11 (21.2)	0.114
Malignancy	1 (0.7)	1 (1.9)	0 (0.0)	0.365	0 (0.0)	1 (1.9)	0.351
Hypertension	29 (19.6)	9 (16.7)	20 (21.3)	0.4964	20 (20.8)	9 (17.3)	0.606
Primary infection focus				<0.001			0.07151
Urinary tract infection	44 (29.9)	6 (11.3)	38 (40.4)		24 (25.0)	20 (39.2)	
Intra-abdominal infection	57 (38.8)	35 (66.0)	22 (23.4)		39 (40.6)	18 (35.3)	
Pneumonia	9 (6.1)	3 (5.7)	6 (6.4)		4 (4.2)	5 (9.8)	
Primary bacteremia	37 (25.2)	9 (17.0)	28 (29.8)		29 (30.2)	8 (15.7)	
Empirical therapy				0.0906			0.371
Monotherapy	83 (56.9)	35 (66.0)	48 (51.6)		56 (59.6)	27 (51.9)	
Combination therapy	63 (43.2)	18 (34.0)	45 (48.4)		38 (40.4)	C25 (48.1)	
Initiation of therapy				0.9855			<0.001
Inappropriate	28 (19.3)	10 (19.2)	18 (19.4)		8 (8.6)	20 (38.5)	
Appropriate	117 (80.7)	42 (80.8)	75 (80.7)		85 (91.4)	32 (61.5)	
Targeted therapy				0.3667			0.222
Monotherapy	60 (42.3)	19 (37.3)	41 (45.1)		35 (38.5)	25 (49.0)	
Combination therapy	82 (57.8)	32 (62.8)	50 (55.0)		56 (61.5)	26 (51.0)	
Clinical outcomes				0.6028			1.000
Cure	134 (90.5)	48 (88.9)	86 (91.5)		87 (90.6)	47 (90.4)	
Death	14 (9.5)	6 (11.1)	8 (8.5)		9 (9.4)	5 (9.6)	
Length of hospitalization, median days (IQR)	11.0 (8.0, 16.0)	11.0 (7.0, 18.0)	12.0 (8.0, 15.0)	0.6743	11.0 (8.0, 17.0)	11.0 (8.0, 15.3)	0.727
Fever	36.9 (36.6, 38.0)	36.8 (36.6, 37.8)	36.9 (36.6, 38.0)	0.616	36.9 (36.6, 37.8)	37.0 (36.7, 38.1)	0.524

*Values are given as N (%) or median (IQR).*

*ESBL+, extended spectrum β-lactamase-producing E. coli; ESBL-, non-extended spectrum β-lactamase-producing E. coli.*

Because several previous studies reported mortality was associated with infections by ESBL+ bacteria (Rottier et al., 2012), we assessed the impact of the ESBL status of isolates on patient outcome (Table 1). Patients with ESBL- and ESBL+ isolates had no differences in major demographic characteristics (*p* > 0.05). More patients with bacteremia due to ESBL+ *E. coli* received inappropriate empirical antibiotic treatment than those with ESBL- *E. coli* (61.5% vs. 91.4%, *p* < 0.001), but these two groups had no significant difference in overall mortality (*p* = 1.000).

### Antimicrobial Resistant Profiles

MIC testing indicated that all isolates were susceptible to ceftazidime/avibactam, carbapenems, and tigecycline (Table 2). One isolate was resistant to polymyxin B. Phenotype testing confirmed that 35.1% of the isolates were ESBL+. The prevalence of ESBL+ was greater in strains from patients associated with UTIs (45.5%) than in strains from patients with other primary sources. Compared with isolates from patients with primary UTIs and IAIs, isolates from patients with primary bacteremia had lower rates of resistance to antibiotics (Table 2). As expected based on a previous study (Peirano and Pitout, 2010), the prevalence of AMR was greater in ST131 than in other STs. In addition, MDR was most common in ST131 (84.2%), ST95 (35.0%), and ST69 (58.3%).

**TABLE 2:** Antimicrobial susceptibility/resistance of *E. coli* isolates.

Antibiotic	***E. coli* (*n* = 148)**					**ESBL+ (*n* = 52)**	**ESBL- (*n* = 96)**	**UTI (*n* = 44)**	**Primary bacteremia (*n* = 37)**	**IAI (*n* = 57)**	**ST69 (*n* = 12)**	**ST95 (*n* = 20)**	**ST131 (*n* = 19)**
	**MIC, range (mg/L)**	**MIC_50_ (mg/L)**	**MIC_90_ (mg/L)**	**R%**	**S%**	R%	R%	R%	R%	R%	R%	R%	R%
ESBL	−	−	−	35.1	−	−	−	45.5	21.6	31.6	16.7	10.0	68.4
Amoxicillin	2–128	128	128	75	20.9	−	61.5	79.5	73	70.2	91.7	60.0	94.7
Amoxicillin/ clavulanic acid	2–128	16	64	37.8	31.1	61.5	25.0	43.2	40.5	29.8	50.0	20.0	31.6
Piperacillin/ tazobactam	0.25–128	4	32	4.1	82.4	7.7	2.1	4.5	5.4	0	16.7	0	0
Cefoperazone/ sulbactam^*a*^	0.25–128	1	16	2	93.2	5.8	0	4.5	2.7	0	0	0	0
Ceftazidime/ avibactam	0.06–4	0.25	1	0	100	0	0	0	0	0	0	0	0
Cefazolin	0.25–128	2	128	37.2	56.8	−	3.1	45.5	24.3	35.1	16.7	10.0	68.4
Cefuroxime	0.25–128	4	128	35.1	61.5	−	2.1	45.5	24.3	29.8	16.7	10.0	73.7
Ceftazidime	0.125–64	0.25	32	18.2	77.7	−	1	18.2	16.2	15.8	8.3	5.0	42.1
Ceftriaxone	0.06–64	0.125	64	33.1	64.9	−	0	40.9	21.6	29.8	8.3	10.0	68.4
Cefepime	0.03–64	0.064	16	12.8	75	−	0	18.2	8.1	10.5	0	0	31.6
Cefoxitin	0.25–128	4	16	9.5	80.4	21.2	3.1	11.4	2.7	10.5	8.3	5.0	0
Moxalactam	0.125–32	0.5	2	0	98.6	0	0	0	0	0	0	0	0
Aztreonam	0.125–64	0.25	64	27	69.6	−	0	29.5	18.9	24.6	8.3	5.0	57.9
Ertapenem	0.007–0.5	0.008	0.064	0	100	0	0	0	0	0	0	0	0
Imipenem	0.06–0.5	0.125	0.25	0	100	0	0	0	0	0	0	0	0
Meropenem	0.015–0.5	0.016	0.064	0	100	0	0	0	0	0	0	0	0
Amikacin	0.5–128	4	8	1.4	98.6	1.9	1.0	2.3	2.7	0	8.3	0	0
Gentamicin	0.25–128	2	128	33.8	66.2	48.1	26	38.6	37.8	24.6	58.3	30.0	42.1
Ciprofloxacin	0.007–32	1	32	56.1	32.4	84.6	40.6	54.5	43.2	63.2	33.3	40.0	78.9
Levofloxacin	0.015–32	1	32	36.5	49.3	73.1	16.7	36.4	24.3	43.9	16.7	5.0	63.2
Trimethoprin/ sulfamethoxzole	0.06–8	0.125	8	43.2	56.8	57.7	35.4	43.2	48.6	35.1	58.3	40.0	57.9
Fosfomycin	0.5–256	0.5	1	2.0	97.3	5.8	0	2.3	0	1.8	0	0	0
Polymyxin B	0.25–4	0.5	1	0.7	99.3	0	1	0	0	1.8	0	0	0
Tigecycline	0.125–0.5	0.25	0.25	0	100	0	0	0	0	0	0	0	0

*UTI, urinarytract infection; BSI, blood stream infection; IAI, intra-abdominal infection; R%, percent resistance; S% percent sensitive. ^*a*^Criteria from Clinical and Laboratory Standards Institute [CLSI] (2020) for cefoperazone were used for cefoperazone-sulbactam.*

**FIGURE 1:**
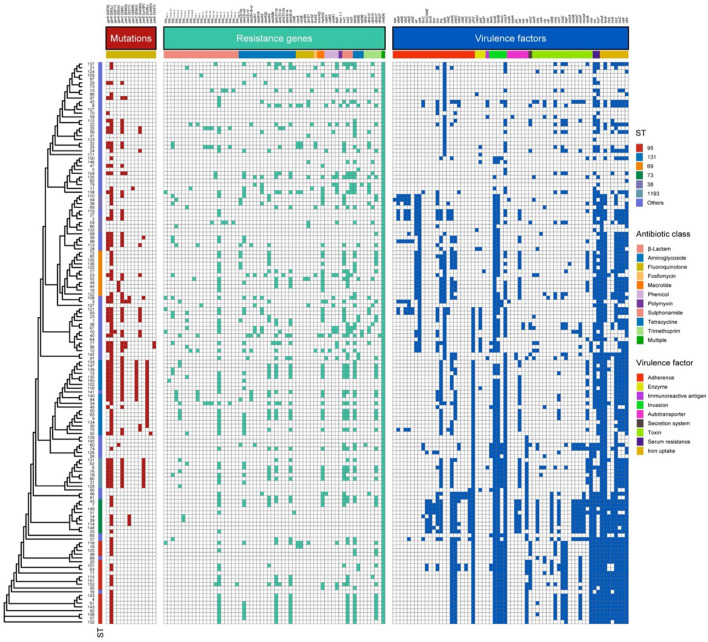
Overall similarity of the 148 *E. coli* isolates from patients with EC-COBSI based on ST and genomic AMR status. Each line shows the strain number, sequence type (ST), mutations conferring fluoroquinolone resistance, and presence of AMR genes and virulence genes in the genome assembly. Red, presence of mutation; Green, presence of AMR gene; Blue, presence of virulence gene; Blank, absent.

We further analyzed the molecular AMR profiles of the 148 *E. coli* isolates (Figure 1 and Supplementary Table 1). This analysis indicated that 38 of the 52 (73.1%) ESBL+ isolates had at least one ESBL gene. The most common ESBL gene was *bla_*CTX*__–__*M*__–__14_*, which occurred in 21.2% of isolates (11/52). There were also 41 other unique resistance genes among a range of STs (Figure 1). Mutations in quinolone resistance-determining regions (QRDRs) were present in 74.3% of the isolates, and the most common variants were S83L in *gyrA* and S80I in *parC* (Supplementary Table 2). Remarkably, one isolate was resistant to polymyxin and was positive for the *mcr-1.1* gene.

We also observed that the prevalence of AMR genes varied according to the primary infection source. In particular, some gene variants were more common in isolates from patients with UTIs (*sul1*, *bla_*TEM*__–1B_*, and *bla_*CTX*__–__*M*__–__55_*; Supplementary Table 1). Among the different STs, the presence of genes conferring resistance to aminoglycoside and fluoroquinolone were found in ST131 (Supplementary Table 1).

### Virulence Genes

In total, we identified 66 virulence genes (Figure 1), and these genes had several different functions. The most common genes functioned in invasion, iron uptake, and serum resistance (Supplementary Table 3). Compared with isolates from patients with UTIs and primary bacteremia, isolates from patients with IAIs had lower frequencies of virulence genes, such as genes connected with adherence (*papC*), invasion (*kpsE*, *kpsM*), and iron uptake (*fyuA*, *ireA*; Supplementary Table 3). Notably, ST95 isolates had a higher frequency of some virulence genes, such as those associated with adherence (*papA*, *papC*), invasion (*neuC*), and toxin production (*cma*, *cvaC*, *hlyF*, *usp*), than ST131 and ST69 isolates.

### Population Structure of *E. coli* Isolates

We successfully sequenced all 148 isolates and identified 59 different STs, including five new STs (Supplementary Table 4). The five most common STs were ST95, ST131, ST69, ST73, and ST1193, and these accounted 51.3% of the total (Supplementary Table 4). ST95 was most common in patients with UTIs (22.7%), ST131 was the most common in patients with IAIs (12.3%), and ST69 was the most common in patients with primary bacteremia (16.2%). There was greater diversity of STs in isolates from patients with IAIs than in isolates from patients with UTIs and primary bacteremia.

**FIGURE 2:**
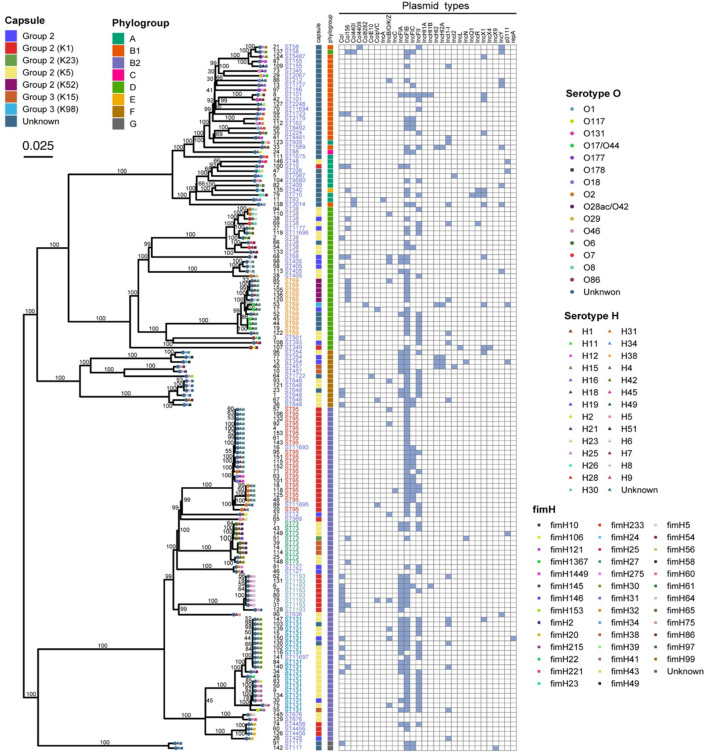
Maximum likelihood phylogenic analysis of 148 *E. coli* isolates from patients with EC-COBSI based on SNPs.

The ML phylogenetic tree indicated two major clades: one clade (Figure 2, top) contained 76 isolates and 44 STs, including ST69; the other clade (Figure 2, bottom) contained 72 isolates and 15 STs, including ST95 and ST131. Phylogroup B2 (47.3%, 70/148) was predominant and in lower clade, and phylogroups D, B1, F, A, G, C, and E were less common and in upper clade, except for G which was in the lower clade. Analysis of encapsulation indicated that 106 (71.6%) isolates had at least one capsule, capsules were most prevalent in group 2 (66.2%), and 41 isolates (27.7%) had K5-specific capsules (Figure 2). We also performed serotype analysis, although most of the isolates (71.6%, 106/148) were unsuccessfully serotyped. We identified 14 serotypes in 42 isolates, and O2 and O6 were the main serotypes. Subtyping of the *fimH* gene showed 40 *fimH* types, but 10 were in untyped isolates. The *fimH27* allele was present in 35 (25.4%) of the typed isolates followed by *fimH30* allele (10.9%). All ST69 and 90.0% (18/20) of ST95 isolates carried *fimH27* allele, and 52.6% (10/19) of ST131 had the *fimH30* allele (*H30*). Eight *E. coli H30* ST131 isolates with resistance to fluoroquinolones (defined as *H30*-R) harbored *bla_*CTX*__–__*M*_*, including two isolates with *bla_*CTX*__–__*M*__–__14_*, two with *bla_*CTX*__–__*M*__–__15_* (defined as *H30*-Rx), two with *bla_*CTX*__–__*M*__–__27_*, one with *bla_*CTX*__–__*M*__–__55_*, and one with *bla_*CTX*__–__*M*__–__137_*.

**FIGURE 3:**
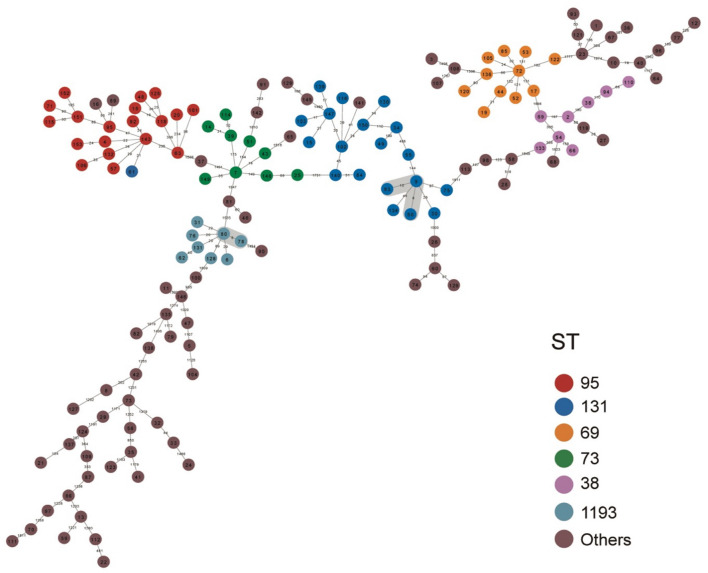
Minimum spanning tree based on the core genome of 148 *E. coli* isolates from patients with EC-COBSI. Colors indicate the classical multilocus STs. According to the framework for interpreting SNP analysis from the FDA-CFSAN, isolates were considered closely related based on the likelihood they arose from the same source when assessing the SNP results. A SNP distance of 10 or less to the nearest neighbor was considered presumptive transmission.

We also constructed a minimum spanning tree based on the core genome to analyze the relationships of the 148 isolates (Figure 3). Based on a predefined cut-off value (distance ≤ 10 SNPs), we predicted 3 presumptive transmissions, one by isolates of ST1193 and two by isolates of ST131. However, these STs indicated no geographic, familial, or other relationships according the patients’ medical records. We also observed high diversity between individual isolates in all branches, which may suggest no dissemination from a common source and no clonal spread among patients.

### Pathogenicity Genes in the Main Sequence Types

We identified 30 different plasmid replicons in 21 incompatibility (Inc) groups (Supplementary Table 5). IncFIB (70.9%, 105/148) and IncFII (38.5%, 57/148) were the most common. IncFIC plasmids were common in ST95 (50.0%, 10/20), and IncFII and IncFIA were common in ST131 (89.9 and 72.2%, respectively). A broader range of col156 plasmids were in ST69 (41.7%), than in ST131 and ST95.

**FIGURE 4:**
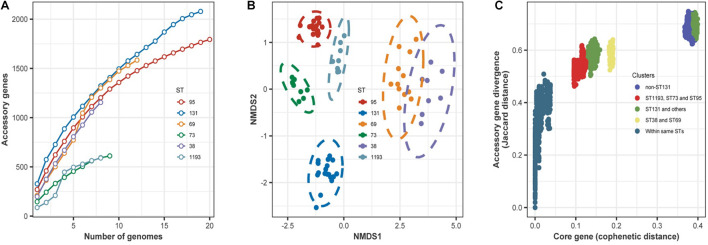
**(A)** Accumulation of accessory genes (*y*-axis) as a function of the number of sampled genomes (*x*-axis) in the dominant STs. For the six different STs, there was a steady increase in the number of new genes identified with increasing sampling, and there was no evidence of saturation in any ST. These accumulation curves fit Heaps’ law. The derived constant α was less than 1 for all STs, indicating an open pangenome. **(B)** Non-metric multidimensional scaling plots of the dominant STs based on shared accessory genes. Dotted lines are 95% confidence limits around the centroid for each ST. **(C)** Comparison of core and accessory genomes of the dominant STs. Each circle is the core genome similarity, plotted as cophenetic distance (*x*-axis) as a function of accessory gene similarity (*y*-axis, Jaccard distance) for each paired comparison within the different groups indicated in the key. Intra-ST comparisons of isolates (dark blue dots, bottom left) indicated little difference in core gene content, with clustering by the origin. In contrast, pairwise comparisons between all isolates of different STs other than ST131 (bright blue dots, top right) form a separate cluster. This discontinuity reflects a distinct clonal structure of the population, because a higher rate of recombination between STs can generate a more homogeneous distribution. Comparison of ST131 with all other isolates indicated two clusters (green dots). Comparison of ST73, ST95, and ST1193 indicated more similarity in core gene divergence and accessory gene content (red dots).

For the main STs in this study, we hypothesized that the accessory genes of the isolates would include genes important in establishing BSI. To measure the size of the accessory genomes, we therefore determined the numbers of new genes identified with increasing numbers of sampled genomes for the dominant STs (Figure 4A). The results indicated the accumulation curve rose more rapidly for ST131 than the other STs.

Then, we determined whether the accessory gene composition was related to ST or some other characteristic(s) of the isolates. We therefore analyzed the similarity between the accessory gene content of each isolate using NMDS based on the Jaccard distance of accessory genes between isolates (Figure 4B). The results showed that the isolates grouped together according to ST. However, there were no significant differences between ST69 and ST38, meaning some accessory genes were shared by these STs.

We further assessed variations in the accessory gene pool by comparing core genome divergence and accessory gene content using pairwise comparisons (Figure 4C). These results indicated that isolates within the same ST had little difference in core gene content. Comparison of ST131 with other STs indicated two clusters: one cluster (Figure 4C, top right) highlighted the divergence of ST131 from the other STs; another cluster (Figure 4C, middle) had accessory gene content that apparently underwent exchange with some STs. Accessory gene content was more divergent between these groups than in the intra-strain comparison, but not as extreme as between ST131 and the other STs. Although ST38 and ST69 were in the same clade in the ML phylogenetic tree (Figure 2), accessory genes in these STs had more divergence than in ST73, ST95, and ST1193.

**FIGURE 5:**
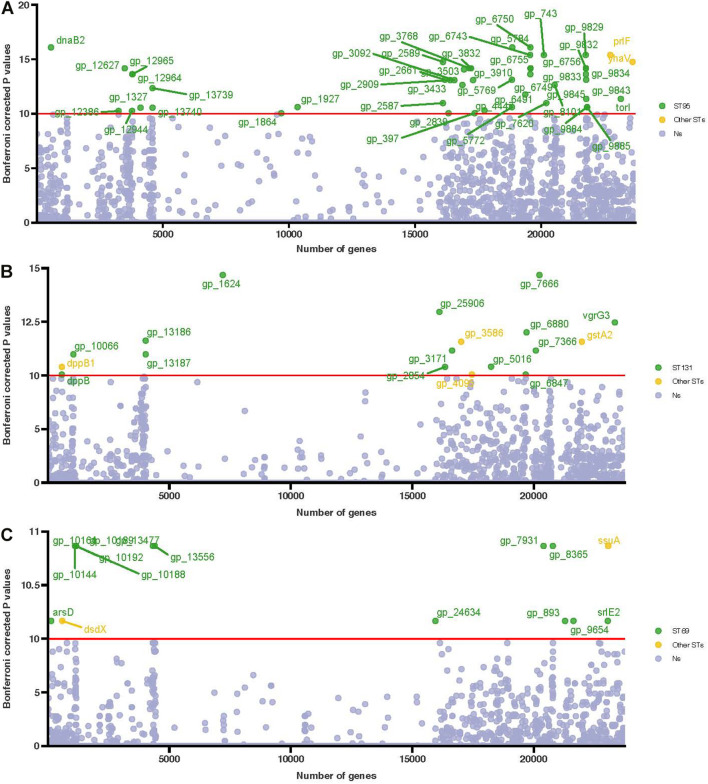
Manhattan plot of the association of genes in the three main STs (ST95, ST131, and ST69) based on genome-wide association analysis. (**A**) ST95 compared with other STs, (**B**) ST131 compared with other STs, and (**C**) ST69 compared with other STs. Association of genes with phenotypes was determined using the multiple Kruskal-Wallis test. Uncorrected *P*-values are shown, and the Bonferroni corrected *P*-values for significance are indicated by the red horizontal lines. Genes more abundant in these three STs are in green, and genes more abundant in other STs are in yellow.

We performed a genome-wide association analysis to identify the determinants of the three most common STs (ST69, ST95, and ST131) and to identify the prevalences of different genes in these STs (Figure 5). Compared with ST69 and ST131, ST95 had some significant associations of the lysozyme gene with the Omptin family outer membrane protease gene (Figure 5A and Supplementary Table 6-1). However, we found no significant associations in genes related to antimicrobial resistance in ST131, although some genes, such as gp-1624 (related to the lipopolysaccharide (LPS) O-antigen length regulator) and *vgrG*3 (related to the type VI secretion system) were associated with ST131 (Figure 5B and Supplementary Table 6-2). Most genes in ST69 were significantly associated with different hypothetical proteins (Figure 5C and Supplementary Table 6-3).

## Discussion

We performed an exploratory analysis of the association of patient characteristics with microbiological determinants in a cohort of patients with EC-COBSI. The results demonstrated that the mortality rate from EC-COBSI in our study population was unrelated to ESBL positivity, and that isolates from IAIs were more genetically diverse than those from the other sources. There was a segregation of STs in the population structure of COBSI.

To our knowledge, this is the first report of mortality from EC-COBSI in China. The present study indicated the in-hospital all-cause 30-day mortality of patients with EC-COBSI was 9.5%, lower than reported other studies (Richelsen et al., 2020), but similar to a study in England (Blandy et al., 2019). These differences in mortality might be due to differences in study design, patient characteristics, primary sources of infection, circulating regional strains, STs of *E. coli*, and treatment strategies. Notably, the present study found no difference in the mortality of patients with infections by ESBL+ *E. coli* or ESBL- *E. coli*, although more patients with ESBL- *E. coli* received appropriate antimicrobials. Thus, the previous findings of a lower mortality rate in patients infected by ESBL- *E. coli* may be because these patients were more likely to receive appropriate antibiotics (Paterson et al., 2004). It should be noted that prompt review and treatment adjustment within 48 h after blood culture may minimize the impact of inappropriate therapy regardless of ESBL positivity (Fitzpatrick et al., 2016). In our setting, the standard use of blood cultures with a rapid reporting system has been used for many years, and this enables clinicians to better adjust therapy in a timely manner and improve patient outcomes.

We confirmed that *bla_*CTX*__–__*M*__–__14_*, *bla_*CTX*__–__*M*__–__27_*, and *bla_*CTX*__–__*M*__–__55_* were the main ESBL genes. There is emerging evidence that *E. coli* STs that cause extraintestinal infections may be from a food or animal reservoir (Sun et al., 2010; Zhang et al., 2015). Our minimum spanning tree analysis also predicted 3 presumptive transmissions. Because relationships due to geography or other features of healthcare could not explain these transmissions, it is possible that these patients were exposed a common source in the food chain. We identified one polymyxin B-resistant *E. coli* that was positive for the *mcr*-*1.1* gene in an elderly patient living in an urban setting who had no history of receiving healthcare. It is therefore not surprising that polymyxin B-resistant *E. coli* may have become regionally or nationally disseminated since the first report of *mcr-1*-positive *E. coli* in China during 2015 (Liu et al., 2016).

Most of our patients had BSI that were secondary to IAIs. In contrast, other studies reported that UTIs were the leading source for EC-COBSI (Bonten et al., 2021). Another Chinese report showed that IAI was the most common source of EC-COBSI (Xiao et al., 2019). This difference was also reflected in the greater diversity of STs in our patients who had IAIs. We speculate that some *E. coli* isolates may translocate from the host gut to the bloodstream. Several previous studies of sepsis patients suggested that infectious *E. coli* strains may have translocated *via* the gut epithelium, because the same *E. coli* strains were also present in high numbers in the guts of these patients (Moreno et al., 2008; Owrangi et al., 2018). Our finding that isolates from patients with IAIs had lower AMR supported this speculation. In general, the strains from patients with IAIs had significantly fewer virulence genes than those from patients with UTIs or primary bacteremia. This is logical, because enteric bacteria may be inadvertently transferred from the gut to the bloodstream during an IAI. This means that isolates that otherwise have relatively low pathogenicity nevertheless can cause BSI, such as ST648 and ST1193.

This study found a segregation of STs, in that some STs with greater antimicrobial resistance were less virulent. Although, ST95 and ST131 were the predominant STs, ST95 isolates had a higher frequency of virulence genes than ST131. On the contrary, ST131 has more antibiotic resistance than ST95 due to plasmids. Previous research found that since the emergence of multi-drug-resistant clades *H30*R and *H30*Rx in North America (Stoesser et al., 2016), these clades have successfully disseminated worldwide (Schaufler et al., 2016). The prevalence of the *H30* clade and the *H30*Rx subclade among *E. coli* ST131 isolates was similar to previous studies (Lafolie et al., 2014; Li et al., 2017). Because we isolated these strains from CO-BSI patients, this important clade and subclade may be endemic in the community. In this study, IncFIA and IncFII were more common in ST131 than ST95, and this was the reason for the MDR phenotype in ST131 (Kallonen et al., 2017). This segregation of STs in the population structure of COBSI caused by *E. coli* supports the hypothesis that these EC-COBSI represent a spill-over of *E. coli* that occupies a commensal niche in the wider human population (Kallonen et al., 2017).

Our accessory gene accumulation curves indicated that ST95, ST131, and ST69 were more likely to mutate than other STs, meaning that these three STs may more easily adapt to different ecological niches. Another study reported no “common sets” of accessory genes in the accessory genome of *E. coli* that would be important in producing bacteremia (Goswami et al., 2018). This agrees with our finding that the accessory gene content was more diverse between different STs than within a single ST. The marked divergence of the accessory genome in the main STs indicated there was little recombination among the different STs. Taken together, this also supports the hypothesis that a primary factor shaping the ST structure of EC-COBSI was competition within the gut commensal niche.

We performed a genome-wide association study to identify genetic determinants that were associated with major STs. Omptins are a family of proteases embedded in the outer membrane that are recognized as key virulence factors for these pathogens because they inactivate host antimicrobial peptides (AMP) and evade AMP killing (Stumpe et al., 1998; Thomassin et al., 2012; Brannon et al., 2015). Our results indicated that the *ompT* gene (encoding omptinT) was strongly associated with ST95. Some genes encoding important proteins were also associated with ST131. Previous studies found that the type VI secretion system (T6SS) has contractile function, and is important in pathogen-host interactions because it injects toxins into bacterial or fungal cells (Trunk et al., 2018; Hu et al., 2021). The LPS O-antigen is also crucial for the virulence of pathogens, and the length of the O-antigen plays a key role in interactions with the complement system (Grossman et al., 1987). Proteins that regulate the chain-length of the O-antigen also have important biological effects (Murray et al., 2006). Thus, all these genes may contribute to the pathogenicity of ST131.

There are some limitations in our study. First, this was a single center-study. Second, we did not investigate the community (non-medical) environments of the different patients. A previous study found that the living environment may play a role in the persistence and transmission of ESBL- *E. coli* in the community (Leverstein-van Hall et al., 2011). The identification of non-healthcare-associated risk factors may contribute to the increasing drug resistance rate among isolates responsible for community-acquired infections. We also did not investigate possibly related cases because this was a retrospective observational study. Third, we did not analyze the risk factors for EC-COBSI due to small study size and lack of stratification. Lastly, although about one-quarter of our patients had bacteremia before hospitalization, and we had no information on previous infections and could not confirm if the index infection was due to recurrence or re-infection.

## Conclusion

The mortality rate from EC-COBSI in our study population was unrelated to ESBL positivity, and most of our isolates had low resistance to most antimicrobial agents. Nonetheless, the *E. coli* isolates in our patients were diverse, and we found that the multiple strains were related. Therefore, interventions that aim to prevent and control EC-COBSI should be implemented at multiple levels that target colonization of patients and the environment.

## Data Availability Statement

The datasets presented in this study can be found in online repositories. The names of the repository/repositories and accession number(s) can be found in the article/Supplementary Material.

## Ethics Statement

The studies involving human participants were reviewed and approved by this work was approved by the Committee on Human Research, Publications and Ethics of The First Hospital of Putian City (No. 2021-004). Written informed consent for participation was not required for this study in accordance with the national legislation and the institutional requirements.

## Author Contributions

FC analyzed the data and drafted the manuscript. TL and AC performed the experiments. YpX and YhX revised the analyzed the data. YC designed and revised the manuscript. FC and TL contributed equally to this work. All authors contributed and approved the final article.

## Conflict of Interest

The authors declare that the research was conducted in the absence of any commercial or financial relationships that could be construed as a potential conflict of interest.

## Publisher’s Note

All claims expressed in this article are solely those of the authors and do not necessarily represent those of their affiliated organizations, or those of the publisher, the editors and the reviewers. Any product that may be evaluated in this article, or claim that may be made by its manufacturer, is not guaranteed or endorsed by the publisher.

## Footnotes

1
http://www.eucast.org/clinical_break-points
