# Supplementary material for: Clinical Characteristics of Patients and Whole Genome Sequencing-Based Surveillance of Escherichia coli Community-Onset Bloodstream Infections at a Non-tertiary Hospital in CHINA
Source: Front Microbiol. 2021 Oct 7;12:748471. doi: 10.3389/fmicb.2021.748471 (PMC8529152; doi:10.3389/fmicb.2021.748471)
Supplement: Supplementary file 1 [file Data_Sheet_1.docx]

Supplementary table 1 Distribution of antimicrobial resistance genes in *E. coli*

| Genes | Total | | UTI | | IAI | | BSI | | p | ST69 | | ST95 | | ST131 | | p | ESBL+E. coli | | ESBL-E. coli | | p |
| --- | --- | --- | --- | --- | --- | --- | --- | --- | --- | --- | --- | --- | --- | --- | --- | --- | --- | --- | --- | --- | --- |
|  | N=148 | % | n=44 | % | n=57 | % | n=37 | % |  | n=12 | % | n=20 | % | n=19 | % |  | n=52 | % | n=96 | % |  |
| *bla_CMY-2_* | 5 | 3.4 | 0 | 0 | 2 | 3.5 | 2 | 5.4 | 0.190 | 0 | 0 | 1 | 5.0 | 0 | 0 | 0.386 | 3 | 5.8 | 2 | 2.1 | 0.479 |
| *bla_CTX-M-137_* | 1 | 0.7 | 0 | 0 | 1 | 1.8 | 0 | 0 | 0.411 | 0 | 0 | 0 | 0 | 1 | 5.3 | 0.366 | 1 | 1.9 | 0 | 0 | 0.755 |
| *bla_CTX-M-14_* | 14 | 9.5 | 7 | 15.9 | 4 | 7.0 | 1 | 2.7 | 0.090 | 1 | 8.3 | 0 | 0 | 2 | 10.5 | 0.207 | 11 | 21.2 | 3 | 3.1 | 0.001 |
| *bla_CTX-M-15_* | 6 | 4.1 | 3 | 6.8 | 0 | 0 | 3 | 8.1 | 0.036 | 0 | 0 | 0 | 0 | 2 | 10.5 | 0.129 | 5 | 9.6 | 1 | 1.0 | 0.037 |
| *bla_CTX-M-27_* | 11 | 7.4 | 5 | 11.4 | 3 | 5.3 | 2 | 5.4 | 0.466 | 0 | 0 | 0 | 0 | 6 | 31.6 | 0.001 | 10 | 19.2 | 1 | 1.0 | 0.000 |
| *bla_CTX-M-3_* | 2 | 1.4 | 1 | 2.3 | 0 | 0 | 1 | 2.7 | 0.338 | 0 | 0 | 0 | 0 | 0 | 0 | NA | 2 | 3.8 | 0 | 0 | 0.234 |
| *bla_CTX-M-55_* | 9 | 6.1 | 5 | 11.4 | 4 | 7.0 | 0 | 0 | 0.040 | 0 | 0 | 0 | 0 | 2 | 10.5 | 0.129 | 7 | 13.5 | 2 | 2.1 | 0.016 |
| *bla_CTX-M-65_* | 3 | 2 | 0 | 0 | 2 | 3.5 | 1 | 2.7 | 0.304 | 0 | 0 | 0 | 0 | 0 | 0 | NA | 2 | 3.8 | 1 | 1.0 | 0.586 |
| *bla_DHA-1_* | 1 | 0.7 | 0 | 0 | 0 | 0 | 1 | 2.7 | 0.265 | 1 | 8.3 | 0 | 0 | 0 | 0 | 0.228 | 0 | 0 | 1 | 1.0 | 1.000 |
| *bla_OXA-1_* | 7 | 4.7 | 4 | 9.1 | 1 | 1.8 | 2 | 5.4 | 0.230 | 0 | 0 | 0 | 0 | 2 | 10.5 | 0.129 | 6 | 11.5 | 1 | 1.0 | 0.014 |
| *bla_OXA-10_* | 2 | 1.4 | 1 | 2.3 | 1 | 1.8 | 0 | 0 | 0.524 | 0 | 0 | 0 | 0 | 0 | 0 | NA | 0 | 0 | 2 | 2.1 | 0.762 |
| *bla_TEM-106_* | 3 | 2.0 | 1 | 2.3 | 2 | 3.5 | 0 | 0 | 0.362 | 0 | 0 | 0 | 0 | 1 | 5.3 | 0.366 | 2 | 3.8 | 1 | 1.0 | 0.586 |
| *bla_TEM-126_* | 3 | 2.0 | 1 | 2.3 | 2 | 3.5 | 0 | 0 | 0.362 | 0 | 0 | 0 | 0 | 1 | 5.3 | 0.366 | 2 | 3.8 | 1 | 1.0 | 0.586 |
| *bla_TEM-135_* | 5 | 3.4 | 1 | 2.3 | 4 | 7.0 | 0 | 0 | 0.106 | 0 | 0 | 0 | 0 | 1 | 5.3 | 0.366 | 3 | 5.8 | 2 | 2.1 | 0.479 |
| *bla_TEM-1A_* | 4 | 2.7 | 1 | 2.3 | 3 | 5.3 | 0 | 0 | 0.206 | 0 | 0 | 1 | 5.0 | 0 | 0 | 0.386 | 0 | 0 | 4 | 4.2 | 0.336 |
| *bla_TEM-1B_* | 73 | 49.3 | 28 | 63.6 | 21 | 36.8 | 18 | 48.6 | 0.028 | 9 | 75.0 | 15 | 75.0 | 13 | 68.4 | 0.878 | 24 | 46.2 | 49 | 51.0 | 0.570 |
| *bla_TEM-1C_* | 2 | 1.4 | 0 | 0 | 2 | 3.5 | 0 | 0 | 0.167 | 0 | 0 | 0 | 0 | 0 | 0 | NA | 1 | 1.9 | 1 | 1.0 | 1.000 |
| *bla_TEM-20_* | 2 | 1.4 | 0 | 0 | 2 | 3.5 | 0 | 0 | 0.167 | 0 | 0 | 0 | 0 | 0 | 0 | NA | 1 | 1.9 | 1 | 1.0 | 1.000 |
| *bla_TEM-220_* | 3 | 2.0 | 1 | 2.3 | 2 | 3.5 | 0 | 0 | 0.362 | 0 | 0 | 0 | 0 | 1 | 5.3 | 0.366 | 2 | 3.8 | 1 | 1.0 | 0.586 |
| *bla_TEM-32_* | 2 | 1.4 | 0 | 0 | 2 | 3.5 | 0 | 0 | 0.167 | 0 | 0 | 0 | 0 | 0 | 0 | NA | 1 | 1.9 | 1 | 1.0 | 1.000 |
| *bla_TEM-79_* | 1 | 0.7 | 1 | 2.3 | 0 | 0 | 0 | 0 | 0.316 | 0 | 0 | 1 | 5.0 | 0 | 0 | 0.386 | 0 | 0 | 1 | 1.0 | 1.000 |
| *aac(3)-IIa* | 10 | 6.8 | 5 | 11.4 | 0 | 0 | 5 | 13.5 | 0.004 | 4 | 33.3 | 0 | 0 | 1 | 5.3 | 0.008 | 5 | 9.6 | 5 | 5.2 | 0.499 |
| *aac(3)-IId* | 37 | 25 | 13 | 29.5 | 12 | 21.1 | 8 | 21.6 | 0.568 | 1 | 8.3 | 6 | 30.0 | 9 | 47.4 | 0.073 | 14 | 26.9 | 23 | 24 | 0.691 |
| *aac(3)-IV* | 3 | 2.0 | 2 | 4.5 | 0 | 0 | 1 | 2.7 | 0.179 | 1 | 8.3 | 0 | 0 | 0 | 0 | 0.228 | 2 | 3.8 | 1 | 1.0 | 0.586 |
| *aac(6')-Ib-cr* | 7 | 4.7 | 4 | 9.1 | 1 | 1.8 | 2 | 5.4 | 0.230 | 0 | 0 | 0 | 0 | 2 | 10.5 | 0.129 | 6 | 11.5 | 1 | 1.0 | 0.014 |
| *aadA1* | 9 | 6.1 | 2 | 4.5 | 4 | 7.0 | 2 | 5.4 | 0.864 | 1 | 8.3 | 0 | 0 | 0 | 0 | 0.228 | 5 | 9.6 | 4 | 4.2 | 0.335 |
| *aadA16* | 1 | 0.7 | 1 | 2.3 | 0 | 0 | 0 | 0 | 0.316 | 0 | 0 | 0 | 0 | 0 | 0 | NA | 1 | 1.9 | 0 | 0 | 0.755 |
| *aadA2* | 13 | 8.8 | 3 | 6.8 | 6 | 10.5 | 3 | 8.1 | 0.797 | 0 | 0 | 0 | 0 | 0 | 0 | NA | 8 | 15.4 | 5 | 5.2 | 0.074 |
| *aadA2b* | 2 | 1.4 | 1 | 2.3 | 1 | 1.8 | 0 | 0 | 0.524 | 1 | 8.3 | 0 | 0 | 0 | 0 | 0.228 | 1 | 1.9 | 1 | 1.0 | 1.000 |
| *aadA5* | 35 | 23.6 | 16 | 36.4 | 9 | 15.8 | 9 | 24.3 | 0.059 | 5 | 41.7 | 2 | 10.0 | 10 | 52.6 | 0.015 | 16 | 30.8 | 19 | 19.8 | 0.194 |
| *aadA8b* | 1 | 0.7 | 0 | 0 | 0 | 0 | 0 | 0 | NA | 0 | 0 | 0 | 0 | 0 | 0 | NA | 1 | 1.9 | 0 | 0 | 0.755 |
| *aph(3'')-Ib* | 48 | 32.4 | 18 | 40.9 | 14 | 24.6 | 11 | 29.7 | 0.208 | 4 | 33.3 | 12 | 60.0 | 8 | 42.1 | 0.295 | 19 | 36.5 | 29 | 30.2 | 0.432 |
| *aph(3')-Ia* | 10 | 6.8 | 2 | 4.5 | 7 | 12.3 | 0 | 0 | 0.020 | 0 | 0 | 1 | 5.0 | 1 | 5.3 | 0.577 | 5 | 9.6 | 5 | 5.2 | 0.499 |
| *aph(3')-IIa* | 3 | 2.0 | 2 | 4.5 | 1 | 1.8 | 0 | 0 | 0.277 | 1 | 8.3 | 0 | 0 | 0 | 0 | 0.228 | 2 | 3.8 | 1 | 1.0 | 0.586 |
| *aph(4)-Ia* | 2 | 1.4 | 1 | 2.3 | 0 | 0 | 1 | 2.7 | 0.338 | 0 | 0 | 0 | 0 | 0 | 0 | NA | 1 | 1.9 | 1 | 1.0 | 1.000 |
| *aph(6)-Id* | 48 | 32.4 | 18 | 40.9 | 15 | 26.3 | 10 | 27 | 0.238 | 4 | 33.3 | 12 | 60.0 | 9 | 47.4 | 0.338 | 19 | 36.5 | 29 | 30.2 | 0.432 |
| *rmtB* | 2 | 1.4 | 1 | 2.3 | 1 | 1.8 | 0 | 0 | 0.524 | 0 | 0 | 0 | 0 | 0 | 0 | NA | 2 | 3.8 | 0 | 0 | 0.234 |
| *oqxA* | 5 | 3.4 | 0 | 0 | 3 | 5.3 | 1 | 2.7 | 0.173 | 0 | 0 | 2 | 10.0 | 0 | 0 | 0.144 | 2 | 3.8 | 3 | 3.1 | 1.000 |
| *oqxB* | 5 | 3.4 | 0 | 0 | 3 | 5.3 | 1 | 2.7 | 0.173 | 0 | 0 | 2 | 10.0 | 0 | 0 | 0.144 | 2 | 3.8 | 3 | 3.1 | 1.000 |
| *qnrB4* | 1 | 0.7 | 0 | 0 | 0 | 0 | 1 | 2.7 | 0.265 | 1 | 8.3 | 0 | 0 | 0 | 0 | 0.228 | 0 | 0 | 1 | 1 | 1.000 |
| *qnrS1* | 11 | 7.4 | 1 | 2.3 | 6 | 10.5 | 4 | 10.8 | 0.175 | 1 | 8.3 | 1 | 5.0 | 0 | 0 | 0.359 | 0 | 0 | 11 | 11.5 | 0.027 |
| *qnrS2* | 3 | 2.0 | 0 | 0 | 3 | 5.3 | 0 | 0 | 0.067 | 0 | 0 | 0 | 0 | 0 | 0 | NA | 3 | 5.8 | 0 | 0 | 0.077 |
| *fosA3* | 5 | 3.4 | 2 | 4.5 | 2 | 3.5 | 1 | 2.7 | 0.905 | 1 | 8.3 | 0 | 0 | 0 | 0 | 0.228 | 3 | 5.8 | 2 | 2.1 | 0.479 |
| *erm(42)* | 1 | 0.7 | 1 | 2.3 | 0 | 0 | 0 | 0 | 0.316 | 0 | 0 | 0 | 0 | 0 | 0 | NA | 1 | 1.9 | 0 | 0 | 0.755 |
| *mph(A)* | 48 | 32.4 | 18 | 40.9 | 12 | 21.1 | 15 | 40.5 | 0.052 | 9 | 75 | 2 | 10.0 | 10 | 52.6 | 0.001 | 21 | 40.4 | 27 | 28.1 | 0.181 |
| *catA1* | 5 | 3.4 | 1 | 2.3 | 2 | 3.5 | 2 | 5.4 | 0.756 | 0 | 0 | 0 | 0 | 0 | 0 | NA | 2 | 3.8 | 3 | 3.1 | 1.000 |
| *catB3* | 3 | 2.0 | 1 | 2.3 | 1 | 1.8 | 1 | 2.7 | 0.952 | 0 | 0 | 0 | 0 | 0 | 0 | NA | 2 | 3.8 | 1 | 1.0 | 0.586 |
| *cmlA1* | 10 | 6.8 | 3 | 6.8 | 5 | 8.8 | 1 | 2.7 | 0.459 | 1 | 8.3 | 0 | 0 | 0 | 0 | 0.228 | 5 | 9.6 | 5 | 5.2 | 0.499 |
| *floR* | 21 | 14.2 | 4 | 9.1 | 12 | 21.1 | 4 | 10.8 | 0.181 | 1 | 8.3 | 3 | 15.0 | 0 | 0 | 0.119 | 11 | 21.2 | 10 | 10.4 | 0.074 |
| *mcr-1.1* | 1 | 0.7 | 0 | 0 | 1 | 1.8 | 0 | 0 | 0.411 | 0 | 0 | 0 | 0 | 0 | 0 | NA | 0 | 0 | 1 | 1.0 | 1.000 |
| *sul1* | 45 | 30.4 | 19 | 43.2 | 11 | 19.3 | 14 | 37.8 | 0.025 | 6 | 50 | 2 | 10.0 | 10 | 52.6 | 0.010 | 20 | 38.5 | 25 | 26.0 | 0.117 |
| *sul2* | 52 | 35.1 | 18 | 40.9 | 18 | 31.6 | 11 | 29.7 | 0.501 | 5 | 41.7 | 12 | 60.0 | 9 | 47.4 | 0.558 | 21 | 40.4 | 31 | 32.3 | 0.325 |
| *sul3* | 11 | 7.4 | 1 | 2.3 | 8 | 14.0 | 1 | 2.7 | 0.034 | 1 | 8.3 | 0 | 0 | 0 | 0 | 0.228 | 7 | 13.5 | 4 | 4.2 | 0.084 |
| *tet(A)* | 64 | 43.2 | 16 | 36.4 | 26 | 45.6 | 18 | 48.6 | 0.493 | 6 | 50 | 12 | 60.0 | 11 | 57.9 | 0.853 | 26 | 50 | 38 | 39.6 | 0.222 |
| *tet(B)* | 11 | 7.4 | 4 | 9.1 | 4 | 7.0 | 2 | 5.4 | 0.813 | 1 | 8.3 | 2 | 10.0 | 0 | 0 | 0.231 | 4 | 7.7 | 7 | 7.3 | 1.000 |
| *tet(M)* | 3 | 2.0 | 1 | 2.3 | 1 | 1.8 | 1 | 2.7 | 0.952 | 0 | 0 | 0 | 0 | 0 | 0 | NA | 3 | 5.8 | 0 | 0 | 0.077 |
| *dfrA1* | 1 | 0.7 | 0 | 0 | 0 | 0 | 1 | 2.7 | 0.265 | 0 | 0 | 0 | 0 | 0 | 0 | NA | 0 | 0 | 1 | 1.0 | 1.000 |
| *dfrA12* | 15 | 10.1 | 3 | 6.8 | 7 | 12.3 | 3 | 8.1 | 0.617 | 0 | 0 | 0 | 0 | 0 | 0 | NA | 10 | 19.2 | 5 | 5.2 | 0.007 |
| *dfrA14* | 10 | 6.8 | 2 | 4.5 | 6 | 10.5 | 2 | 5.4 | 0.459 | 1 | 8.3 | 1 | 5.0 | 0 | 0 | 0.359 | 0 | 0 | 10 | 10.4 | 0.039 |
| *dfrA17* | 48 | 32.4 | 19 | 43.2 | 14 | 24.6 | 13 | 35.1 | 0.139 | 5 | 41.7 | 12 | 60.0 | 10 | 52.6 | 0.603 | 18 | 34.6 | 30 | 31.3 | 0.676 |
| *dfrA27* | 1 | 0.7 | 1 | 2.3 | 0 | 0 | 0 | 0 | 0.316 | 0 | 0 | 0 | 0 | 0 | 0 | NA | 1 | 1.9 | 0 | 0 | 0.755 |
| *mdf(A)* | 148 | 100 | 44 | 100 | 57 | 100 | 37 | 100 | NA | 12 | 100 | 20 | 100 | 19 | 100 | NA | 52 | 100 | 96 | 100 | NA |

Abbreviation: UTI, urinary tract infection; IAI, intra-abdominal infection; BSI, bloodstream infection; ESBL+*E. coli*, ESBL-producing *E. coli*; ESBL-*E. coli*, non-ESBL-producing *E. coli*.

Supplementary table 2 Distribution of mutations with quinolone resistance determining regions(QRDRs)

| DNA gyrase/topoisomerase IV genes mutations | | | | | | |
| --- | --- | --- | --- | --- | --- | --- |
|  | *gyrA* | No.of number | *parC* | No.of number | *parE* | No.of number |
|  | S83L | 57/148(38.5%) | S80I | 38/148(25.7%) | I529L | 17/148(11.5%) |
|  | S83A | 4/148(2.7%) | S80I E84V | 11/148(7.4%) | S458A | 12/148(8.1%) |
|  | S83L D87N | 45/148(30.4%) | S80I E84G | 4/148(2.7%) | L416F | 10/148(6.8%) |
|  | S83L D87Y | 1/148(0.7%) | S80I S57T | 1/148(0.7%) | L445H | 1/148(0.7%) |
|  |  |  | S80I E84G S57T | 1/148(0.7%) | I355T | 2/148(1.4%) |
|  |  |  |  |  | I529L S458A | 1/148(0.7%) |
| Total | 107/148(72.3%) | | 55/148(37.2%) | | 43/148(29.1%) | |

Supplementary table 3 Frequency of virulence-associated genes in *E. coli* isolated by primary infection sources and STs

| Function | Genes | Total (N=148) | UTI（n=44) | IAI（n=57) | Bacteremia（n=37) | p | ST69(n=12) | ST95(n=20) | ST131(n=19) | p |
| --- | --- | --- | --- | --- | --- | --- | --- | --- | --- | --- |
|  |  | % | % | % | % |  | % | % | % |  |
| Adherence | | | | | | | | | | |
|  | *aap* | 0.7 | 0 | 0 | 2.7 | 0.265 | 0 | 0 | 0.0 | NA |
|  | *afaA* | 7.4 | 13.6 | 3.5 | 8.1 | 0.170 | 0 | 0 | 5.3 | 0.366 |
|  | *afaB* | 2.7 | 4.5 | 1.8 | 2.7 | 0.713 | 0 | 0 | 0.0 | NA |
|  | *afaC* | 7.4 | 13.6 | 3.5 | 8.1 | 0.170 | 0 | 0 | 5.3 | 0.366 |
|  | *afaD* | 9.5 | 13.6 | 8.8 | 8.1 | 0.657 | 0 | 0 | 5.3 | 0.366 |
|  | *afaE* | 2.0 | 4.5 | 0 | 2.7 | 0.179 | 0 | 0 | 0.0 | NA |
|  | *air* | 19.6 | 18.2 | 10.5 | 29.7 | 0.061 | 100 | 0 | 0.0 | 0.000 |
|  | *eilA* | 28.4 | 31.8 | 21.1 | 32.4 | 0.359 | 100 | 0 | 0.0 | 0.000 |
|  | *focC* | 4.7 | 2.3 | 7.0 | 5.4 | 0.521 | 0 | 0 | 0.0 | NA |
|  | *focC*/*sfaE* | 7.4 | 6.8 | 1.8 | 13.5 | 0.072 | 0 | 10 | 0.0 | 0.144 |
|  | *focG* | 6.1 | 6.8 | 1.8 | 10.8 | 0.152 | 0 | 0 | 0.0 | NA |
|  | *focI* | 3.4 | 2.3 | 1.8 | 8.1 | 0.278 | 0 | 0 | 0.0 | NA |
|  | *hra* | 30.4 | 38.6 | 19.3 | 35.1 | 0.076 | 16.7 | 10.0 | 15.8 | 0.817 |
|  | *iha* | 38.5 | 38.6 | 26.3 | 51.4 | 0.047 | 75.0 | 0 | 94.7 | 0.000 |
|  | *lpfA* | 35.8 | 29.5 | 43.9 | 29.7 | 0.228 | 100 | 0 | 5.3 | 0.000 |
|  | *nfaE* | 4.7 | 9.1 | 1.8 | 5.4 | 0.230 | 0 | 0 | 5.3 | 0.366 |
|  | *papA* | 52 | 59.1 | 36.8 | 59.5 | 0.035 | 83.3 | 100 | 63.2 | 0.003 |
|  | *papC* | 41.2 | 59.1 | 17.5 | 45.9 | 0.000 | 66.7 | 100 | 47.4 | 0.001 |
|  | *sfaD* | 8.8 | 6.8 | 7.0 | 10.8 | 0.769 | 0 | 10 | 0.0 | 0.144 |
|  | *sfaE* | 1.4 | 2.3 | 0 | 2.7 | 0.338 | 0 | 0 | 0.0 | NA |
|  | *sfaS* | 2.7 | 4.5 | 0 | 2.7 | 0.179 | 0 | 10 | 0.0 | 0.144 |
|  | *tcpC* | 8.8 | 9.1 | 5.3 | 13.5 | 0.383 | 0 | 0 | 0.0 | NA |
|  | *yfcV* | 55.4 | 65.9 | 43.9 | 59.5 | 0.072 | 0 | 100 | 100.0 | 0.000 |
| Enzyme | | | | | | | | | | |
|  | *capU* | 2.0 | 0 | 3.5 | 2.7 | 0.304 | 0 | 0 | 0.0 | NA |
|  | *gad* | 10.8 | 6.8 | 14.0 | 10.8 | 0.498 | 0 | 0 | 5.3 | 0.366 |
|  | *katP* | 0.7 | 0 | 1.8 | 0 | 0.411 | 8.3 | 0 | 0.0 | 0.228 |
| Immunoreactive antigen | | | | | | | | | | |
|  | *cba* | 3.4 | 0 | 5.3 | 5.4 | 0.140 | 0 | 0 | 0 | NA |
| Invasion | | | | | | | | | | |
|  | *ibeA* | 7.4 | 4.5 | 3.5 | 13.9 | 0.163 | 0 | 10 | 5.3 | 0.371 |
|  | *kpsE* | 77.0 | 88.6 | 57.9 | 91.7 | 0.000 | 100 | 100 | 89.5 | 0.129 |
|  | *kpsM* | 70.9 | 86.4 | 52.6 | 83.3 | 0.000 | 66.7 | 100 | 89.5 | 0.012 |
|  | *neuC* | 25.7 | 31.8 | 21.1 | 25.0 | 0.461 | 0 | 100 | 0.0 | 0.000 |
|  | *ompT* | 73.0 | 77.3 | 68.4 | 75.0 | 0.612 | 66.7 | 100 | 100.0 | 0.002 |
| Autotransporter | | | | | | | | | | |
|  | *eatA* | 1.4 | 0 | 1.8 | 2.7 | 0.439 | 0 | 0 | 5.3 | NA |
|  | *epeA* | 0.7 | 0 | 1.8 | 0 | 0.411 | 0 | 0 | 89.5 | NA |
|  | *pic* | 7.4 | 6.8 | 3.5 | 13.5 | 0.201 | 0 | 0 | 89.5 | NA |
|  | *sat* | 32.4 | 36.4 | 17.5 | 45.9 | 0.010 | 83.3 | 0 | 0.0 | 0.000 |
|  | *tsh* | 1.4 | 0 | 3.5 | 0 | 0.167 | 0 | 0 | 100.0 | NA |
|  | *vat* | 28.4 | 31.8 | 19.3 | 37.8 | 0.122 | 0 | 70 | 0 | 0.000 |
| Secretion system | | | | | | | | | | |
|  | *etsC* | 14.2 | 15.9 | 15.8 | 8.1 | 0.505 | 0 | 60 | 0 | 0.000 |
| Toxin | | | | | | | | | | |
|  | *astA* | 6.1 | 2.3 | 7.0 | 8.1 | 0.416 | 8.3 | 0 | 0.0 | 0.228 |
|  | *cea* | 14.2 | 11.4 | 8.8 | 21.6 | 0.180 | 8.3 | 10.0 | 0.0 | 0.231 |
|  | *celb* | 2.0 | 0 | 3.5 | 2.7 | 0.304 | 0 | 0 | 0.0 | NA |
|  | *cia* | 7.4 | 6.8 | 8.8 | 8.1 | 0.936 | 8.3 | 10.0 | 5.3 | 0.852 |
|  | *cib* | 1.4 | 2.3 | 0 | 0 | 0.316 | 0 | 5 | 0.0 | 0.386 |
|  | *clbB* | 10.8 | 15.9 | 3.5 | 13.5 | 0.067 | 0 | 20.0 | 0.0 | 0.018 |
|  | *cma* | 18.9 | 11.4 | 24.6 | 16.2 | 0.219 | 8.3 | 60.0 | 0.0 | 0.000 |
|  | *cnf1* | 12.8 | 18.2 | 3.5 | 18.9 | 0.030 | 0 | 10 | 31.6 | 0.022 |
|  | *cvaC* | 24.3 | 27.3 | 26.3 | 13.5 | 0.263 | 8.3 | 95.0 | 0.0 | 0.000 |
|  | *hlyF* | 25.7 | 29.5 | 24.6 | 21.6 | 0.705 | 16.7 | 100 | 0.0 | 0.000 |
|  | *mcbA* | 0.7 | 0 | 0 | 2.7 | 0.253 | 8.3 | 0 | 0.0 | 0.228 |
|  | *mchB* | 9.5 | 6.8 | 7.0 | 16.2 | 0.289 | 0 | 0 | 0.0 | NA |
|  | *mchC* | 9.5 | 6.8 | 7.0 | 16.2 | 0.289 | 0 | 0 | 0.0 | NA |
|  | *mchF* | 25.0 | 31.8 | 19.3 | 24.3 | 0.354 | 0 | 70.0 | 0.0 | 0.000 |
|  | *mcmA* | 10.1 | 9.1 | 5.3 | 18.9 | 0.111 | 0 | 0 | 0.0 | NA |
|  | *senB* | 26.4 | 34.1 | 19.3 | 27.0 | 0.242 | 66.7 | 0 | 63.2 | 0.000 |
|  | *usp* | 34.5 | 40.9 | 21.1 | 43.2 | 0.036 | 0 | 100 | 0.0 | 0.000 |
| Serum resistance | | | | | | | | | | |
|  | *iss* | 67.6 | 70.5 | 56.1 | 78.4 | 0.067 | 91.7 | 100 | 63.2 | 0.002 |
|  | *traT* | 65.5 | 77.3 | 64.9 | 51.4 | 0.051 | 75.0 | 95.0 | 73.7 | 0.123 |
| Iron uptake | | | | | | | | | | |
|  | *chuA* | 77.0 | 88.6 | 57.9 | 89.2 | 0.000 | 100 | 100 | 100.0 | NA |
|  | *fyuA* | 74.3 | 90.9 | 56.1 | 81.1 | 0.000 | 100 | 100 | 100.0 | NA |
|  | *ireA* | 23.6 | 36.4 | 10.5 | 27.0 | 0.006 | 8.3 | 90.0 | 10.5 | 0.000 |
|  | *iroN* | 32.4 | 34.1 | 31.6 | 29.7 | 0.914 | 16.7 | 85.0 | 0.0 | 0.000 |
|  | *irp2* | 73.0 | 90.9 | 54.4 | 78.4 | 0.000 | 100 | 100 | 89.5 | 0.129 |
|  | *iucC* | 66.2 | 75.0 | 54.4 | 67.6 | 0.090 | 91.7 | 100 | 89.5 | 0.207 |
|  | *iutA* | 67.6 | 75.0 | 54.4 | 73.0 | 0.055 | 91.7 | 100 | 89.5 | 0.207 |
|  | *sitA* | 76.4 | 88.6 | 64.9 | 75.7 | 0.023 | 75 | 100 | 100.0 | 0.009 |

Supplementary table 4 Constitution and distribution of STs of *E. coli*

| ST | Total | | UTI | | IAI | | Primary bacteremia | | Other or unknown sources | |
| --- | --- | --- | --- | --- | --- | --- | --- | --- | --- | --- |
|  | n | % | n | % | n | % | n | % | n | % |
| 95 | 20 | 13.5 | 10 | 22.7 | 3 | 5.3 | 4 | 10.8 | 3 | 30.0 |
| 131 | 19 | 12.8 | 6 | 13.6 | 7 | 12.3 | 5 | 13.5 | 1 | 10.0 |
| 69 | 12 | 8.1 | 3 | 6.8 | 1 | 1.8 | 6 | 16.2 | 2 | 20.0 |
| 73 | 9 | 6.1 | 3 | 6.8 | 1 | 1.8 | 4 | 10.8 | 1 | 10.0 |
| 38 | 8 | 5.4 | 2 | 4.5 | 2 | 3.5 | 4 | 10.8 | - | - |
| 1193 | 8 | 5.4 | 2 | 4.5 | 4 | 7.0 | 2 | 5.4 | - | - |
| 648 | 6 | 4.1 | 2 | 4.5 | 3 | 5.3 | 1 | 2.7 | - | - |
| 405 | 4 | 2.7 | 3 | 6.8 | 1 | 1.8 | - | - | - | - |
| 354 | 3 | 2 | - | - | 1 | 1.8 | 1 | 2.7 | 1 | 10.0 |
| 4456 | 3 | 2 | - | - | - | - | 3 | 8.1 | - | - |
| 58 | 2 | 1.4 | 1 | 2.3 | 1 | 1.8 | - | - | - | - |
| 101 | 2 | 1.4 | - | - | 2 | 3.5 | - | - | - | - |
| 117 | 2 | 1.4 | - | - | 1 | 1.8 | 1 | 2.7 | - | - |
| 127 | 2 | 1.4 | 1 | 2.3 | - | - | 1 | 2.7 | - | - |
| 155 | 2 | 1.4 | 1 | 2.3 | - | - | 1 | 2.7 | - | - |
| 457 | 2 | 1.4 | 1 | 2.3 | 1 | 1.8 | - | - | - | - |
| 676 | 2 | 1.4 | - | - | 2 | 3.5 | - | - | - | - |
| 10 | 1 | 0.7 | - | - | 1 | 1.8 | - | - | - | - |
| 12 | 1 | 0.7 | - | - | 1 | 1.8 | - | - | - | - |
| 48 | 1 | 0.7 | - | - | - | - | 1 | 2.7 | - | - |
| 68 | 1 | 0.7 | - | - | 1 | 1.8 | - | - | - | - |
| 88 | 1 | 0.7 | 1 | 2.3 | - | - | - | - | - | - |
| 93 | 1 | 0.7 | - | - | 1 | 1.8 | - | - | - | - |
| 156 | 1 | 0.7 | - | - | 1 | 1.8 | - | - | - | - |
| 162 | 1 | 0.7 | - | - | 1 | 1.8 | - | - | - | - |
| 212 | 1 | 0.7 | - | - | 1 | 1.8 | - | - | - | - |
| 224 | 1 | 0.7 | - | - | 1 | 1.8 | - | - | - | - |
| 226 | 1 | 0.7 | - | - | 1 | 1.8 | - | - | - | - |
| 345 | 1 | 0.7 | 1 | 2.3 | - | - | - | - | - | - |
| 349 | 1 | 0.7 | - | - | 1 | 1.8 | - | - | - | - |
| 393 | 1 | 0.7 | - | - | - | - | - | - | 1 | 10.0 |
| 409 | 1 | 0.7 | - | - | 1 | 1.8 | - | - | - | - |
| 428 | 1 | 0.7 | - | - | - | - | 1 | 2.7 | - | - |
| 501 | 1 | 0.7 | - | - | 1 | 1.8 | - | - | - | - |
| 540 | 1 | 0.7 | - | - | 1 | 1.8 | - | - | - | - |
| 569 | 1 | 0.7 | 1 | 2.3 | - | - | - | - | - | - |
| 636 | 1 | 0.7 | - | - | 1 | 1.8 | - | - | - | - |
| 710 | 1 | 0.7 | - | - | - | - | 1 | 2.7 | - | - |
| 939 | 1 | 0.7 | - | - | - | - | 1 | 2.7 | - | - |
| 1177 | 1 | 0.7 | 1 | 2.3 | - | - | - | - | - | - |
| 1589 | 1 | 0.7 | - | - | 1 | 1.8 | - | - | - | - |
| 1675 | 1 | 0.7 | - | - | 1 | 1.8 | - | - | - | - |
| 1722 | 1 | 0.7 | 1 | 2.3 | - | - | - | - | - | - |
| 1723 | 1 | 0.7 | - | - | 1 | 1.8 | - | - | - | - |
| 1727 | 1 | 0.7 | - | - | 1 | 1.8 | - | - | - | - |
| 2067 | 1 | 0.7 | - | - | 1 | 1.8 | - | - | - | - |
| 2179 | 1 | 0.7 | - | - | 1 | 1.8 | - | - | - | - |
| 2248 | 1 | 0.7 | - | - | 1 | 1.8 | - | - | - | - |
| 3014 | 1 | 0.7 | - | - | 1 | 1.8 | - | - | - | - |
| 4481 | 1 | 0.7 | - | - | 1 | 1.8 | - | - | - | - |
| 5487 | 1 | 0.7 | - | - | 1 | 1.8 | - | - | - | - |
| 7087 | 1 | 0.7 | - | - | 1 | 1.8 | - | - | - | - |
| 8492 | 1 | 0.7 | 1 | 2.3 |  |  | - | - | - | - |
| 9580 | 1 | 0.7 | - | - | 1 | 1.8 | - | - | - | - |
| 11693 | 1 | 0.7 | - | - | 1 | 1.8 | - | - | - | - |
| 11694 | 1 | 0.7 | - | - | - | - | - | - | 1 | 10.0 |
| 11695 | 1 | 0.7 | 1 | 2.3 | - | - | - | - | - | - |
| 11696 | 1 | 0.7 | 1 | 2.3 | - | - | - | - | - | - |
| 11697 | 1 | 0.7 | 1 | 2.3 | - | - | - | - | - | - |
| Total | 148 | 100 | 44 | 100 | 57 | 100 | 37 | 100.0 | 10 | 100 |

Supplementary table 5 Plasmid replicons in main STs

|  | Total (N=148) | ST69 (n=12) | ST95 (n=20) | ST131 (n=19) | Other STs (n=97) |
| --- | --- | --- | --- | --- | --- |
| Col | 22 | 0 | 0 | 3 | 19 |
| Col156 | 16 | 5 | 0 | 1 | 10 |
| Col440I | 3 | 0 | 0 | 0 | 3 |
| Col440II | 2 | 0 | 0 | 0 | 2 |
| Col8282 | 1 | 1 | 0 | 0 | 0 |
| ColE10 | 1 | 0 | 0 | 0 | 1 |
| ColpVC | 4 | 1 | 0 | 0 | 3 |
| IncA | 1 | 0 | 0 | 0 | 1 |
| IncB/O/K/Z | 13 | 1 | 2 | 3 | 7 |
| IncC | 1 | 0 | 1 | 0 | 0 |
| IncFIA | 37 | 1 | 0 | 13 | 23 |
| IncFIB | 105 | 11 | 20 | 18 | 56 |
| IncFIC | 25 | 0 | 10 | 0 | 15 |
| IncFII | 57 | 5 | 5 | 16 | 31 |
| IncHI1A | 1 | 0 | 0 | 0 | 1 |
| IncHI1B | 2 | 0 | 0 | 0 | 2 |
| IncHI2 | 5 | 1 | 0 | 0 | 4 |
| IncHI2A | 5 | 1 | 0 | 0 | 4 |
| IncI1-I | 16 | 2 | 0 | 5 | 9 |
| IncI2 | 2 | 0 | 0 | 0 | 2 |
| IncL | 1 | 0 | 0 | 0 | 1 |
| IncN | 2 | 0 | 0 | 0 | 2 |
| IncQ1 | 1 | 0 | 0 | 0 | 1 |
| IncR | 3 | 0 | 0 | 0 | 3 |
| IncX1 | 12 | 1 | 0 | 1 | 10 |
| IncX4 | 1 | 0 | 0 | 0 | 1 |
| IncX9 | 1 | 0 | 0 | 0 | 1 |
| IncY | 9 | 0 | 0 | 0 | 9 |
| p0111 | 5 | 1 | 0 | 0 | 4 |
| repA | 1 | 0 | 0 | 1 | 0 |

Supplementary Table 6-1 ST95-GWAS results

| Gene | Annotation | Odds_ratio | Naive_p | Bonferroni_p |
| --- | --- | --- | --- | --- |
| gp_2909 | anion permease | 378 | 3.44E-18 | 8.18E-14 |
| gp_397 | antirestriction protein | 136.125 | 3.75E-15 | 8.91E-11 |
| gp_2661 | antitermination protein | Inf | 3.18E-18 | 7.56E-14 |
| gp_7620 | antitermination protein | Inf | 4.42E-16 | 1.05E-11 |
| gp_1927 | ASCH domain-containing protein | Inf | 1.03E-15 | 2.45E-11 |
| gp_3768 | DNA-binding transcriptional regulator DsdC | Inf | 2.82E-19 | 6.70E-15 |
| gp_743 | DUF977 family protein | Inf | 1.69E-20 | 4.01E-16 |
| gp_5769 | fimbrial-like protein | Inf | 3.18E-18 | 7.56E-14 |
| gp_9832 | helix-turn-helix domain-containing protein | Inf | 2.82E-19 | 6.70E-15 |
| gp_9885 | helix-turn-helix domain-containing protein | Inf | 1.03E-15 | 2.45E-11 |
| gp_1864 | HNH endonuclease | 136.125 | 3.75E-15 | 8.91E-11 |
| gp_5784 | hypothetical protein | Inf | 3.38E-21 | 8.02E-17 |
| gp_6750 | hypothetical protein | Inf | 3.38E-21 | 8.02E-17 |
| gp_9829 | hypothetical protein | Inf | 1.69E-20 | 4.01E-16 |
| gp_2589 | hypothetical protein | Inf | 7.32E-20 | 1.74E-15 |
| gp_12627 | hypothetical protein | Inf | 2.82E-19 | 6.70E-15 |
| gp_6755 | hypothetical protein | Inf | 2.82E-19 | 6.70E-15 |
| gp_6756 | hypothetical protein | Inf | 2.82E-19 | 6.70E-15 |
| gp_9834 | hypothetical protein | Inf | 2.82E-19 | 6.70E-15 |
| gp_12964 | hypothetical protein | Inf | 9.88E-19 | 2.35E-14 |
| gp_12965 | hypothetical protein | Inf | 9.88E-19 | 2.35E-14 |
| gp_6749 | hypothetical protein | Inf | 9.88E-19 | 2.35E-14 |
| gp_9843 | hypothetical protein | Inf | 3.18E-18 | 7.56E-14 |
| gp_3092 | hypothetical protein | 378 | 3.44E-18 | 8.18E-14 |
| gp_3910 | hypothetical protein | 378 | 3.44E-18 | 8.18E-14 |
| gp_3433 | hypothetical protein | Inf | 9.55E-18 | 2.27E-13 |
| gp_8101 | hypothetical protein | Inf | 9.55E-18 | 2.27E-13 |
| gp_13739 | hypothetical protein | 281.25 | 1.87E-17 | 4.43E-13 |
| gp_6491 | hypothetical protein | Inf | 7.17E-17 | 1.70E-12 |
| gp_2587 | hypothetical protein | Inf | 4.42E-16 | 1.05E-11 |
| gp_5772 | hypothetical protein | Inf | 1.03E-15 | 2.45E-11 |
| gp_9884 | hypothetical protein | Inf | 1.03E-15 | 2.45E-11 |
| gp_13740 | hypothetical protein | 156.8571 | 1.17E-15 | 2.78E-11 |
| gp_4447 | hypothetical protein | 203.8182 | 2.08E-15 | 4.93E-11 |
| gp_12386 | hypothetical protein | Inf | 2.32E-15 | 5.51E-11 |
| gp_3503 | lysozyme | 593.75 | 3.55E-19 | 8.43E-15 |
| gp_12944 | omptin family outer membrane protease OmpT | Inf | 2.32E-15 | 5.51E-11 |
| gp_3832 | Putative defective protein IntQ | Inf | 2.82E-19 | 6.70E-15 |
| gp_1327 | RadC family protein | 156.8571 | 1.17E-15 | 2.78E-11 |
| gp_2839 | recombinase family protein | 136.125 | 3.75E-15 | 8.91E-11 |
| dnaB2 | replicative DNA helicase | Inf | 3.38E-21 | 8.02E-17 |
| torI | Response regulator inhibitor for tor operon | Inf | 1.82E-16 | 4.33E-12 |
| gp_6743 | TerC family protein | Inf | 1.69E-20 | 4.01E-16 |
| gp_9833 | transcriptional regulator | Inf | 9.88E-19 | 2.35E-14 |
| gp_9845 | tyrosine-type recombinase/integrase | Inf | 1.82E-16 | 4.33E-12 |

Supplementary Table 6-2 ST131-GWAS results

| Gene | Annotation | Odds_ratio | Naive_p | Bonferroni_p |
| --- | --- | --- | --- | --- |
| gp_1624 | LPS O-antigen length regulator | Inf | 8.78E-20 | 2.08E-15 |
| gp_7666 | bifunctional DNA-formamidopyrimidine glycosylase/DNA-(apurinic or apyrimidinic site) lyase | Inf | 8.78E-20 | 2.08E-15 |
| gp_25906 | N-acetyltransferase | Inf | 4.59E-18 | 1.09E-13 |
| vgrG3 | type VI secretion system tip protein VgrG | Inf | 1.43E-17 | 3.39E-13 |
| gp_6880 | DUF4123 domain-containing protein | Inf | 4.14E-17 | 9.82E-13 |
| gp_13186 | Alw26I/Eco31I/Esp3I family type II restriction adenine-specific DNA-methyltransferase | 267.75 | 1.01E-16 | 2.39E-12 |
| gp_3171 | dimethyl sulfoxide reductase subunit A | Inf | 2.91E-16 | 6.92E-12 |
| gp_7366 | NADH-dependent methylglyoxal reductase | Inf | 2.91E-16 | 6.92E-12 |
| gp_10066 | hypothetical protein | 212.5 | 4.37E-16 | 1.04E-11 |
| gp_13187 | DNA cytosine methyltransferase | 212.5 | 4.37E-16 | 1.04E-11 |
| gp_5016 | AAA family ATPase | 175.6667 | 1.65E-15 | 3.92E-11 |
| gp_2854 | PstS family phosphate ABC transporter substrate-binding protein | Inf | 1.69E-15 | 4.02E-11 |
| gp_6847 | hypothetical protein | Inf | 3.83E-15 | 9.10E-11 |
| dppB | dipeptide ABC transporter permease DppB | Inf | 3.83E-15 | 9.10E-11 |

Supplementary Table 6-3 ST69-GWAS results

| Gene | Annotation | Odds_ratio | Naive_p | Bonferroni_p |
| --- | --- | --- | --- | --- |
| gp_10144 | hypothetical protein | Inf | 5.74E-16 | 1.36E-11 |
| gp_10161 | hypothetical protein | Inf | 5.74E-16 | 1.36E-11 |
| gp_10164 | hypothetical protein | Inf | 5.74E-16 | 1.36E-11 |
| gp_10188 | tyrosine-type recombinase/integrase | Inf | 5.74E-16 | 1.36E-11 |
| gp_10189 | hypothetical protein | Inf | 5.74E-16 | 1.36E-11 |
| gp_10192 | Gfo/Idh/MocA family oxidoreductase | Inf | 5.74E-16 | 1.36E-11 |
| gp_13477 | hypothetical protein | Inf | 5.74E-16 | 1.36E-11 |
| gp_13556 | hypothetical protein | Inf | 5.74E-16 | 1.36E-11 |
| gp_7931 | aliphatic sulfonate ABC transporter permease SsuC | Inf | 5.74E-16 | 1.36E-11 |
| gp_8365 | hypothetical protein | Inf | 5.74E-16 | 1.36E-11 |
| arsD | Arsenical resistance operon trans-acting repressor ArsD | Inf | 2.87E-15 | 6.81E-11 |
| srlE2 | PTS glucitol/sorbitol transporter subunit IIB | Inf | 2.87E-15 | 6.81E-11 |
| gp_24634 | hypothetical protein | Inf | 2.87E-15 | 6.81E-11 |
| gp_893 | YadA-like family protein | Inf | 2.87E-15 | 6.81E-11 |
| gp_9654 | hypothetical protein | Inf | 2.87E-15 | 6.81E-11 |
